# Genome-wide identification and characterization of UBP gene family in wheat (*Triticum aestivum* L.)

**DOI:** 10.7717/peerj.11594

**Published:** 2021-06-15

**Authors:** Miaoze Xu, Peng Jin, Tingting Liu, Shiqi Gao, Tianye Zhang, Fan Zhang, Xiaolei Han, Long He, Jianping Chen, Jian Yang

**Affiliations:** State Key Laboratory for Quality and Safety of Agro-products, Institute of Plant Virology, Ningbo University, Ningbo, China

**Keywords:** Wheat, UBP, Gene family, Evolutionary patterns, Stress responses, Virus-induced gene silencing (VIGS)

## Abstract

Ubiquitination is essential for plant growth and development. Deubiquitination cooperates with ubiquitination to regulate the ubiquitination levels of target proteins. The ubiquitin-specific protease (UBP) family is the largest group of deubiquitinases (DUBs), which perform extensive and significant roles in eukaryotic organisms. However, the *UBP* genes in wheat (*TaUBPs*) are not identified, and the functions of *TaUBPs* are unknown. The present study identified 97 *UBP* genes in the whole genome of *T*. *aestivum*. These genes were divided into 15 groups and non-randomly distributed on chromosomes of *T*. *aestivum*. Analyses of evolutionary patterns revealed that *TaUBPs* mainly underwent purification selection. The studies of *cis*-acting regulatory elements indicated that they might be involved in response to hormones. Quantitative real-time PCR (qRT-PCR) results showed that *TaUBPs* were differentially expressed in different tissues. Besides, several *TaUBPs* were significantly up-regulated when plants were treated with salicylic acid (SA), implying that these DUBs may play a role in abiotic stress responses in plants and few *TaUBPs* displayed differential expression after viral infection. Furthermore, *TaUBP1A.1* (*TraesCS1A02G432600.1*) silenced by virus-induced gene silencing (VIGS) facilitates *Chinese wheat mosaic virus* (CWMV) infection in wheat, indicating that *TaUBP1A.1* may be involved in a defense mechanism against viruses. This study comprehensively analyzed the *UBP* gene family in wheat and provided a basis for further research of *TaUBPs* functions in wheat plant response to viral infection.

## INTRODUCTION

Post-translational modifications (PTMs), including phosphorylation, ubiquitination, sumoylation, glycosylation and lipidation, control many cellular processes in eukaryotes (Clague, Coulson & Urbe, 2012; Isono & Nagel, 2014). Ubiquitination is one of the most vital PTMs and is involved in diverse cellular pathways and physiological events, such as cell-cycle progression, immune responses, and DNA repair (Pickart, 2004; Vierstra, 2009). Protein ubiquitination and deubiquitination have been widely studied (Frappier & Verrijzer, 2011; Zhang, 2003). Proteins are ubiquitinated through three ordered steps, and a cascade of three enzymes is involved: E1 ubiquitin activating enzyme, E2 ubiquitin conjugating enzyme, and E3 ubiquitin ligase (Atanassov, Koutelou & Dent, 2011; Zhou et al., 2017). The number of conjugated ubiquitin molecules and the type of ubiquitin linkage determine the fortune of ubiquitinated substrate proteins. For a longtime, functional studies of ubiquitylation have focused on the function of E3 ubiquitin ligase that binds the substrate and thus confers specificity. In plants, the multi-subunit E3 ligases comprise the Cullin RING ligases (CRLs) which is important for promoting ubiquitylation. SCF complex is the largest group of CRLs and consists of four subunits: Cullin 1 (CUL1), S-phase kinase-associated protein 1 (SKP1/ASK), F-box substrate-binding protein and the RING subunit RING-box 1 (RBX1). Additionally, a conserved protein complex called the COP9 signalosome (CSN) is required for CRL activity (Wei, Serino & Deng, 2008).

Ubiquitination and deubiquitination coordinate the binding of ubiquitin to its substrate. Deubiquitination gets involved in regulating the ubiquitination levels of target proteins and is critical for regulating cellular processes (Vierstra, 2009; Wilkinson, 2000). For instance, deubiquitination is responsible for the activation of ubiquitin molecules after translation. It is also essential for the recycling of the ubiquitin molecules and can rescue proteins from degradation before they are recognized by the degradation machinery. Additionally, it could affect the binding affinity of the target protein to its interactor protein and thereby regulate downstream processes (Isono & Nagel, 2014). The processes of deubiquitination are regulated by deubiquitinating enzymes (DUBs). By explicitly removing ubiquitin moieties, DUBs deubiquitinate target proteins and affect their activity, stability and fate (Katz, Isasa & Crosas, 2010; Neutzner & Neutzner, 2012). Among the several types of DUBs, ubiquitin-specific proteases are the largest and most diverse subfamily in eukaryotes (Amerik & Hochstrasser, 2004; Nijman et al., 2005; Wilkinson, 1997). Ubiquitin-specific protease is abbreviated as UBP in the plant, Ubp in fungus and USP in human. In the following, it is generally expressed as UBP. UBP proteins are highly conserved and contain a ubiquitin carboxyl-terminal hydrolase (UCH) domain, which commonly possesses two similar triads of catalytic residues: each triad contains highly conserved cysteine (Cys) and histidine (His) residues which are crucial for deubiquitination (Yan et al., 2000). Besides, *UBPs* can regulate the function of E3 ubiquitin ligases. For instance, the deneddylating activity of CSN and/or the deubiquitylating activity of UBP12 maintain CRL levels in plants (Wu, Chan & Chien, 2006). In humans, USP15 coordinates with CSN to remove conjugated ubiquitin chains from the RBX1 subunit of CRL for CRL adapter proteins’ stability (Chou et al., 2017). The deubiquitylating enzymes, USP7 and USP9X, differentially regulate the ubiquitin E3 ligase MARCH7 (Nathan et al., 2008). The inhibition of USP7 leads to the degradation of the E3 ligase MDM2 (Turnbull et al., 2017). The stability of the E3 ubiquitin ligase MARCH6 is regulated by USP19 (Nakamura et al., 2014). Currently, 16 *Ubp* genes in yeast (Wilkinson, 1997), 27 *UBP* genes in *A*. *thaliana* (Yan et al., 2000) and 25 *UBP* genes in rice (Wang et al., 2018a) have been identified. Studies in yeast have revealed several Ubps-controlled processes, such as stress responses, energy metabolism, nutrient utilization and sexual reproduction (Auesukaree et al., 2009; Dudley et al., 2005; Kahana, 2001). In plants, *UBPs* have been shown to be involved in controlling cell proliferation (Du et al., 2014; Liu et al., 2008), endoreplication (Xu et al., 2016), root hair elongation (Doelling et al., 2001), root differentiation (An et al., 2018), flowering time (Derkacheva et al., 2016), pollen development and transmission (Nassrallah et al., 2018), canavanine resistance (Yan et al., 2000), regulation of MYC2 levels in jasmonate responses (Jeong et al., 2017), abscisic acid (ABA)-mediated resistance to salt and drought stress (Zhao et al., 2016), pathogen defense (Ewan et al., 2011; Zhou et al., 2017), and deubiquitination of monoubiquitinated-H2A and -H2B (Derkacheva et al., 2016; Nassrallah et al., 2018; Walton et al., 2016). Therefore, *UBPs* are essential to many important facets of plant growth and development. However, as most research has focused on single *UBP* genes, systematic analyses of *UBP* gene family members are few. Despite the fact that wheat is an important food crop globally, the *UBP* gene family members in wheat, their functions and their evolutionary relationships to other crop species have not yet been reported. As *UBPs* are essential for many agriculturally significant traits, understanding the role of *UBPs* in wheat can be an important tool to improve wheat quality and yield.

Ubiquitination has been reported to improve plant defense against various pathogens (Dreher & Callis, 2007). Previous research has shown that the SCF complex participated in plant-virus interactions. First, restriction of the replication of *Plantago asiatica mosaic virus* and *Potato virus X*, virus-induced necrosis, and the host and non-hosts resistance require an essential SKP1-interacting eukaryotic protein, named SGT1 (Komatsu et al., 2010). Second, *N* gene-mediated resistance to *Tobacco mosaic virus* (TMV) arose due to virus-induced gene silencing of *SKP1*, *SGT1*, or *CSN* in *N*. *benthamiana* (Liu et al., 2002). Third, the F-box protein, ACIF, affects *N* gene-mediated responses to TMV and is indispensable for TMV-triggered hypersensitive response in *Nicotiana tabacum* (Van den Burg et al., 2008). According to previous reports, ubiquitin-specific proteases (USPs) have multiple functions in the immune response against viral infections. USP4 positively regulates RIG-I-mediated antiviral response through deubiquitination and stabilization of RIG-I (Wang et al., 2013). USP15 promotes RIG-I-mediated antiviral signaling by deubiquitylating TRIM25 and negatively regulates virus-induced type I interferon signaling (Pauli et al., 2014; Zhang et al., 2015). USP17 is involved in virus-triggered type I IFN signaling (Chen et al., 2010). In plants, *UBPs* function in regulating immunity (Zhou et al., 2017). For instance, UBP12 and UBP13 in *A*. *thaliana*, as well as their tobacco homologue, NtUBP12, negatively regulate plant immunity (Ewan et al., 2011). The deubiquitinating enzymes, Ubp14 and Ubp4 in *M*. *oryzae*, are required for pathogen virulence (Que et al., 2020; Wang et al., 2018b). To date, the role of *UBPs* in the defense of viral infections has not yet been reported although *UBPs* are critical for plant immunity.

The growth and development of wheat are restrained by abiotic and biotic stresses, such as cold, drought, and plant viruses. Plant RNA viruses are one of the major causes of losses of economically important agriculture (Sanfaçon, 2017). For example, a soil-borne virus disease of wheat caused grain yield losses commonly of 10–30% and sometimes up to 70% in Shandong province, China (Chen, 1993). Among these viruses, CWMV and *wheat yellow mosaic virus* (WYMV), threaten wheat production worldwide. CWMV is a member of the genus *Furovirus*, family *Virgaviridae* (Adams, Antoniw & Kreuze, 2009), and its genome consists of bipartite single-stranded positive-sense RNA, including CWMV RNA1 and CWMV RNA2 (Diao et al., 1999). CWMV RNA1 encodes two replication-related proteins and a movement protein (MP) required for viral movement. It is predicted that CWMV RNA2 encodes four proteins, including a coat protein (CP), two minor CP-related proteins (N-CP and CP-RT), and a cysteine-rich protein (CRP) (Andika et al., 2013; Diao et al., 1999; Sun et al., 2013a; Sun et al., 2013b). WYMV belongs to the genus *Bymovirus*, family *Potyviridae*, and has a genome containing two positive single RNA strands (Zhang et al., 2019). Both WYMV RNA1 and WYMV RNA2 encode a polyprotein, respectively. The polyprotein encoded by RNA1 produces eight proteins, including the CP and the nuclear inclusion b protein (NIb), necessary for virus replication. The polyprotein encoded by RNA2 produces two proteins, P1 and P2. CWMV and/or WYMV induce abnormal growth and development of wheat, and infection with CWMV and/or WYMV dramatically reduce wheat yields. *UBPs* are vital for various plant physiological activities, mainly in plant immunity. However, there is no research on the relationships between *UBPs* in wheat and viral infections. Therefore, we identified the *UBP* gene family in wheat and further explored the roles of wheat *UBP* genes in stress responses.

In this study, we identified 97 *UBP* genes in wheat and analyzed their phylogenetic relationship, evolutionary patterns and divergence patterns. We also found an abundance of hormone-related *cis*-acting regulatory elements in *TaUBPs*. We further analyzed the expression levels of 15 *TaUBPs* in different tissues and examined their expression patterns in response to salicylic acid (SA). Additionally, we examined the *TaUBPs* expression patterns in response to CWMV or WYMV. This work lays a reliable bioinformatic foundation for studies of the *TaUBP* gene family, particularly for investigations of the relationships between *TaUBPs* and viral infections.

### MATERIALS & METHODS

### Identification of *UBP* genes in wheat

To identify *UBP* genes in *T*. *aestivum* (*TaUBPs*), UBP protein sequences in *A*. *thaliana* (AtUBPs) (https://www.arabidopsis.org/) were used as seed sequences to search the wheat database using Ensembl Plants (http://plants.ensembl.org/). According to the filtering conditions (E-val <1.0E−5, %ID ≥ 50), redundant genes were removed, and the longest representative transcripts were selected for more accurate analyses. The potential members of the *TaUBP* gene family were verified using Pfam (https://pfam.xfam.org/) by submitting obtained putative TaUBP protein sequences.

### Characterization of *TaUBPs*

Information about the *TaUBP* gene family, such as chromosomal localization, the CDSs’ length, and the number of amino acids obtained from the Ensembl Plants. The theoretical isoelectric point (pI) and molecular weight (MW) of each TaUBP protein were obtained using ExPAsy (https://web.expasy.org/compute_pi/). Subcellular localization was predicted by Plant-mPLoc (http://www.csbio.sjtu.edu.cn/bioinf/plant-multi/) and the signal peptides of the TaUBP proteins were predicted using SignaIP5.0 (http://www.cbs.dtu.dk/services/SignalP/).

### Multiple sequence alignments and phylogenetic analysis

Three data sets were used for phylogenetic analysis, including identified TaUBP protein sequences, 27 AtUBP protein sequences (Liu et al., 2008) downloaded from TAIR (https://www.arabidopsis.org/), and 25 UBP protein sequences in *O*. *sativa* (OsUBPs) (Wang et al., 2018a) downloaded from the Rice Genome Annotation Project (http://rice.plantbiology.msu.edu/downloads_gad.shtml). Multiple sequence alignments were performed through MEGA-X software using MUSCLE function. Further, the neighbor-joining method was used to generate a phylogenetic tree based on 1000 bootstrap replicates and p-distance methods, which were used with the pairwise deletion option to address gaps in the amino acid sequences (Kumar et al., 2018). Next, using the same methodology, the phylogenetic tree of TaUBP protein sequences was constructed. The genome information was provided in Table S1.

### Analysis of chromosomal location and duplication of *TaUBPs*

The wheat genomic sequences and genome annotation files were downloaded from the Ensembl Plants database. Then, we used them to generate a graph of chromosomal location and detect duplication relationships of *TaUBPs* through TBtools software using Graphics function (Chen et al., 2020).

### Calculation of Ka/Ks values

Ka/Ks values are the ratio of the number of nonsynonymous substitutions per nonsynonymous site (Ka) to the number of synonymous substitutions per synonymous site (Ks), which is a powerful indicator for measuring selection pressure. Generally, if the Ka/Ks value >1, some of the mutations are profitable under advantageous selection; if the Ka/Ks value = 1, the mutations are neutral; if the Ka/Ks value <1, the mutation restrict the purifying selection (Shiu et al., 2004). Ka and Ks values were calculated through TBtools software using Ka/Ks Calculator function. The divergence time (T) was calculated as T = Ks/(2 × 9.1 × 10^−9^) million years ago (Mya) (Hurst, 2002; Yang & Bielawski, 2000).

### *Cis*-acting regulatory elements analysis

The promoter region, 2.0 kb upstream of the transcription start site, of all of the *TaUBPs*, were obtained from the Ensembl Plants database. Then, the *cis*-acting regulatory elements were screened via PlantCARE (http://bioinformatics.psb.ugent.be/webtools/plantcare/html/).

### Plant growth, SA treatment, and inoculation of virus

Seeds of the wheat cultivar, ‘cv Yangmai 158′, were germinated in an artificial growth chamber: 25 ± 2 °C and 70% relative humidity under long-day conditions (16 h light/8 h dark cycles) (Yang et al., 2020). The detailed information on the artificial growth chamber is provided in Table S1. The grown wheat plants at the three leaf-stage were used to analyze gene expression levels in response to salicylic acid (SA) treatment. 18 wheat plants were treated with 100 μM SA solution or distilled water (as controls). Then, all samples were collected at six (0, 1, 3, 6, 12, and 24 h) time intervals from inoculation.

Full-length cDNA clones of CWMV and WYMV RNAs have previously been constructed (Yang et al., 2016; Zhang et al., 2019). ‘cv Yangmai 158′plants at the three leaf-stage were inoculated with inoculation buffer (as controls), CWMV or WYMV. Virus transcription and friction inoculation were performed as previously described (Yang et al., 2020). After inoculation, wheat plants were grown on a mixed soil matrix (peat: vermiculite = 1:1) under a long-day photoperiod, at 15 ± 2 °C and 70% relative humidity. Then, all samples were harvested at 8, 11, 14, and 17 days post-inoculation (dpi), with three biological replicates per sample.

The collected leaf samples were immediately frozen in liquid nitrogen and stored at −80 °C prior to the extraction of total RNA. The experiment was independently repeated three times.

### Gene expression analysis by qRT-PCR

Quantitative real-time PCR (qRT-PCR) was performed to validate the expression levels of *TaUBPs*. Total RNA was isolated for each sample using the R6827 Plant RNA Kit protocol (OMEGA). First-strand cDNA was synthesized from the total RNA using the First Strand cDNA Synthesis Kit ReverTra Ace -α- (TOYOBO). Then, qRT-PCR was carried out using SYBR-green fluorescence and the LightCycler^®^480 Real-Time PCR System (Roche). The procedure used for qRT-PCR was 3 min at 95 °C, followed by 40 cycles of 15 s at 95  °C, 30 s at 62 °C, and 30 s at 72 °C. The *T*. *aestivum cell division cycle* (*TaCDC*) gene (Accession Number: XM_020313450) was used as an internal reference gene (Zhang et al., 2019). The relative expression levels of *TaUBPs* were calculated using the 2^−ΔΔ*Ct*^ method (Livak & Schmittgen, 2001). The detailed information of the instruments and the reagents used in this experiment is provided in Table S1.

### Virus-induced gene silencing (VIGS) in wheat

*Barley stripe mosaic virus* (BSMV) is a positive-sense RNA virus and its genome consists of tripartite single-stranded RNA, including BSMV α, β and γ. BSMV-based gene silencing vectors have been widely used in wheat (Holzberg et al., 2002). We got the best fragment sequence (300 bp) of *TaUBP1A.1* (*TraesCS1A02G432600.1*) for VIGS through an online website (https://solgenomics.net/) using VIGS Tool function and choosing *T*. *aestivum* database. Next, the best fragment was amplified from the cDNA of the wheat plant and then digested with *Pac* I and *Not* I restriction enzymes. The products were inserted into the BSMV γ to generate recombinant plasmid BSMV γ: *TaUBP1A.1*. Then BSMV α, β, γ and γ: *TaUBP1A.1* were digested with *Mlu* I, *Spe* I, *Mlu* I and *BssH* II restriction enzymes. Subsequently, using RiboMAX ^TM^ Large Scale RNA Production Systems-T7 (Promega), *in vitro* transcriptions of linearized plasmid transcripts of BSMV RNA α, β and γ/ γ: *TaUBP1A.1* were obtained, and they were mixed at a molar ratio of 1:1:1. The former (BSMV RNA α, β and γ) was named BSMV: 00 (as negative control), and the latter (BSMV RNA α, β and γ: *TaUBP1A.1*) was named BSMV: *TaUBP1A.1*. Additionally, FES was used as inoculation buffer (0.1 M glycine, 0.06 M potassium phosphate, 1% sodium pyrophosphate decahydrate, 1% bentonite, 1% celite, pH 8.5). Then viruses were inoculated into leaves of three-leaf-stage wheat plants. In the same way, *in vitro* transcriptions of linearized plasmid transcripts of CWMV RNA R1 and R2 were also mixed at a molar ratio of 1:1, then inoculated into the upper leaves of the BSMV-infected wheat plants. The detailed information of the reagents used in this experiment is provided in Table S1.

## Results

### Genome-wide identification and characterization of the *UBP* gene family in wheat

After genome-wide searching of *UBP* homologs, a total of 97 candidate *UBP* genes in wheat were identified. Then, to verify the UCH domain’s existence, the 97 candidate UBP protein sequences were submitted to the Pfam database. All 97 candidate UBP proteins were found to contain the UCH domain, suggesting that they are *UBP* gene family members (*TaUBPs*) (Fig. S1). The detailed characteristics of *TaUBPs*, including the Ensembl wheat gene ID, chromosome location, the number of exons, the CDSs’ length, the number of amino acids, physicochemical parameters, predicted subcellular localization, and the presence of signal peptide, are provided in Table S2. The number of exons ranged from 2 to 32, and the CDSs’ length ranged from 366 to 4023 bp. Corresponding to the CDSs’ length, protein size varies significantly, as the number of encoded amino acids ranged from 121 to 1340 aa. The largest protein *TaUBP1D.1* (*TraesCS1D02G441600.1*) was 11 times larger than the smallest *TaUBP5D.1* (*TraesCS5D02G214000.1*). The predicted MW of TaUBP proteins varied from 13.28 to 152.71 kDa and the theoretical pI ranged from 4.45 to 9.60. Based on these findings, individual proteins belonging to the *TaUBP* gene family possess different physicochemical properties in wheat.

To understand the function of the identified *TaUBPs*, we predicted the subcellular localization and signal peptides. The prediction results showed that 94 *TaUBPs* exhibited nuclear localization, and *TaUBP1A.1*, *TaUBP1B.1* (*TraesCS1B02G468200.1*) and *TaUBP1D.2* (*TraesCS1D02G441900.1*) were located in both the nucleus and the chloroplast. The results of signal peptide prediction revealed that *TaUBP5A.1* (*TraesCS5A02G246000.3*), *TaUBP5B.1* (*TraesCS5B02G243400.1*) and *TaUBP5D.2* (*TraesCS5D02G252600.2*) contained a signal peptide each (Table S2).

### Phylogenetic analysis

**Figure 1:**
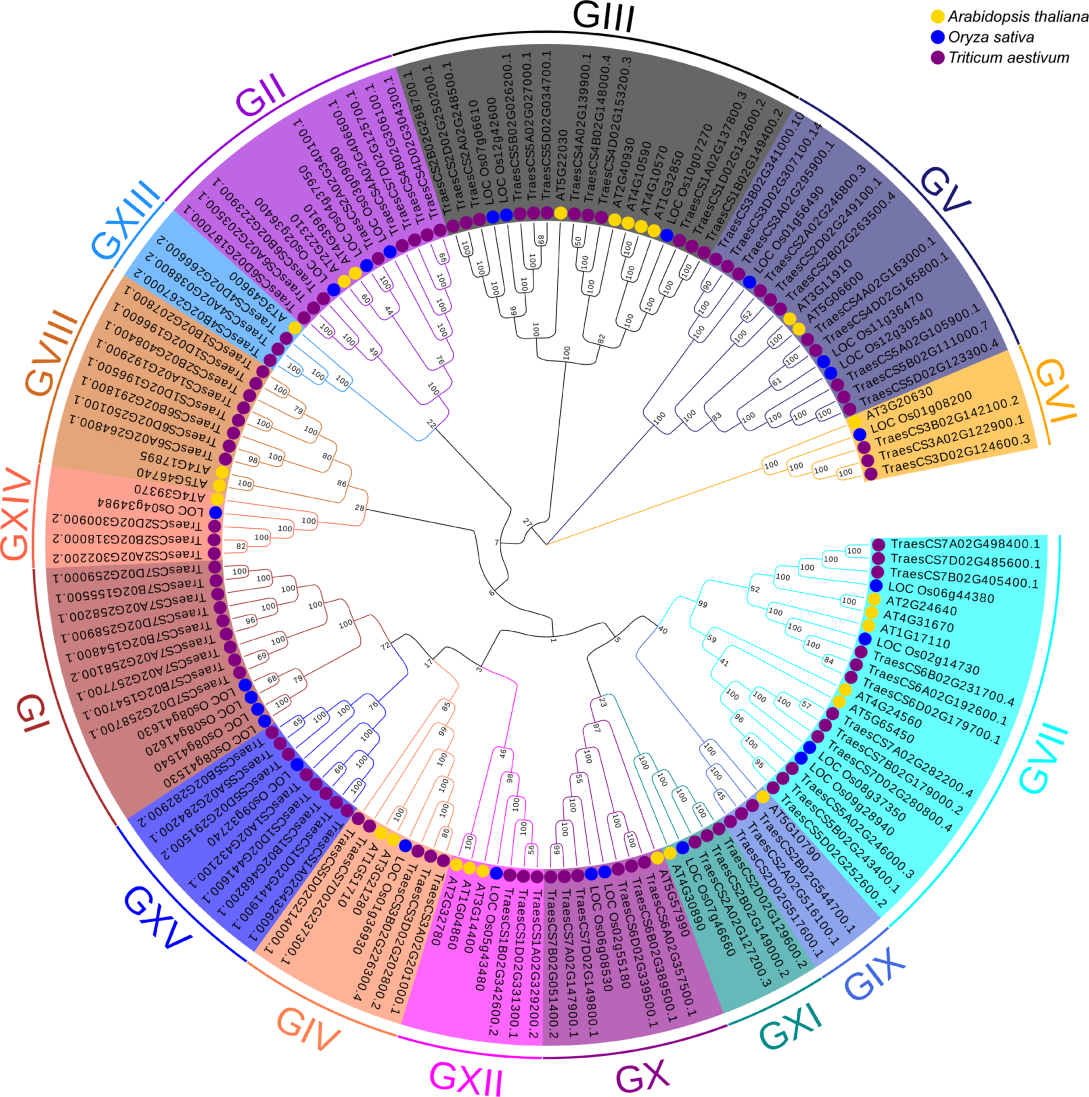
The phylogenetic tree based on alignment of UBP proteins from *A*. *thaliana*, *O*. *sativa* and *T*.* aestivum*. The phylogenetic tree was generated by the p-distance methods using MEGA X software with 1000 bootstrap replicates. All *UBPs* are divided into 15 subclasses represented by specific colored backgrounds. *AtUBPs*, *OsUBPs* and *TaUBPs* are indicated by gold dots, blue dots and purple dots, respectively.

To better understand the evolutionary relationships and to classify *TaUBPs*, a phylogenetic tree was constructed using 27 AtUBP, 25 OsUBP and 97 TaUBP protein sequences (Fig. 1). We also constructed a phylogenetic tree using 97 TaUBP protein sequences (Fig. S2). According to two phylogenetic trees and sequence similarity of all UBP proteins, the 97 *TaUBPs* were clustered into 15 groups (G1-G15). As illustrated in Fig. 1, the phylogenetic tree showed that TaUBP proteins shared high homology with AtUBP and OsUBP proteins. There are 8 *TaUBPs* in group G15, and protein domain analysis showed that TaUBP1A.1, TaUBP1B.1, TaUBP1D.1 and TaUBP1D.2 proteins had a DUF4220 domain each, as well as TaUBP1A.1, TaUBP1A.2 (*TraesCS1A02G432100.1*), TaUBP1B.1, TaUBP1D.1 and TaUBP1D.2 proteins had a DUF594 domain each (Fig. S1). DUF4220 and DUF594 domains are unique and do not exist in AtUBP and OsUBP proteins (Wu et al., 2019). In addition, *UBP* genes from the same species often exist in pairs, such as *TaUBP1A.3* (*TraesCS1A02G192900.1*) and *TaUBP1D.3* (*TraesCS1D02G196500.1*), implying that they are paralogous genes. There are some closely related gene pairs from different species, such as *TaUBP2A.1* (*TraesCS2A02G340100.1*) and *OsUBP04g.1* (*LOC_Os04g37950.1*), suggesting that they may be orthologous. Moreover, *TaUBPs* existed in each group, and most of the groups contained *TaUBPs*, *AtUBPs* and *OsUBPs*.

### Visualization of chromosomal location and duplication of *TaUBPs*

**Figure 2:**
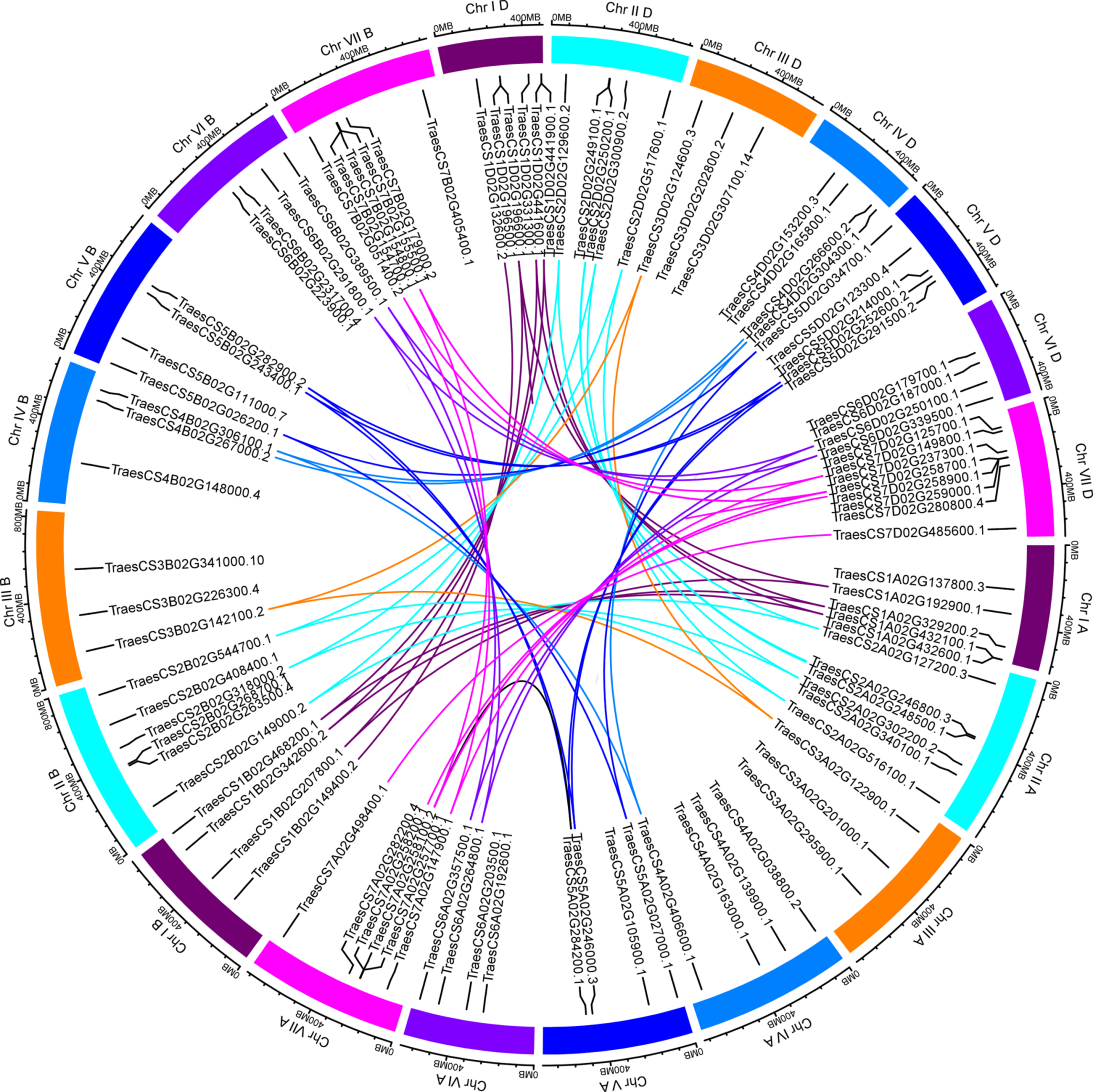
Distribution and duplication events of 97 *TaUBPs*. The paralogous *TaUBP* gene pairs mapped to 21 wheat chromosomes. Chromosome number is indicated outside the outer circle. The duplication events of different chromosomes are highlighted with different colored lines inside. The scale is in mega-bases (Mb).

Based on the available wheat genome annotation information, a total of 97 *TaUBPs* were mapped onto 21 wheat chromosomes to further investigate their functions (Table S2). Chromosomal locations were detected using TBtools, and the visualized distribution of *TaUBPs* is shown in Fig. 2. There were 32, 30, and *35 TaUBPs* non-randomly distributed in the A, B, and D sub-genomes, respectively. The number of *TaUBPs* per chromosome varied from a minimum of three genes to a maximum of eight genes. Within the sub-genome D, chromosome 7 had eight *TaUBPs*, whereas chromosomes 3A, 3B, 3D*,* and 4B had only three *TaUBPs* each. Within all sub-genomes, eight wheat chromosomes contained four *TaUBPs* each, five wheat chromosomes contained six *TaUBPs* each, and three wheat chromosomes contained five *TaUBPs* each. Hence, the chromosomal distribution of *TaUBPs* was scattered and non-random.

It has been confirmed that tandem and segmental duplications are two main causes of gene family expansion in plants (Cannon et al., 2004). According to the chromosomal location map (Fig. 2), 97 *TaUBPs* were distributed irregularly across 21 chromosomes. Moreover, the duplication relationships of *TaUBPs* among the A, B, and D sub-genomes were analyzed. As illustrated in Fig. 2, we identified two tandem duplication clusters and 54 collinear *TaUBP* gene pairs. Two groups of two tandem duplicated genes were located in chromosomes 1D and 7D. Additionally, 54 collinear *TaUBP* gene pairs were distributed in different chromosomes.

### Evolutionary and divergence patterns

The Ka/Ks ratio is an indicator of selective pressure acting on a protein-coding gene. To determine the selection mode of duplicated *UBP* genes, Ka/Ks ratios were calculated for each gene pair (Table S3). The results illustrated that the Ka/Ks ratios of the 48 orthologous genes (*T. aestivum*-*O*. *sativa*, *Ta*-*Os*) varied from 0.032 to 0.652 (Table S3), indicating that these *UBP* genes had been influenced principally by the high purifying selection. The Ka/Ks ratios of the 54 paralogous genes (*T. aestivum*-*T. aestivum*, *Ta*-*Ta*) were all less than one, and the two paralogous genes were 1.001 and 1.090 (Table S3).

The divergence time (T) was calculated, revealing that the 56 paralogous genes (*Ta*-*Ta*) diverged between 1.057 and 68.418 million years ago (Mya), and that the 48 orthologous genes (*Ta*-*Os*) were estimated to have diverged between 22.478 and 73.254 Mya (Table S3).

### Prediction of *cis*-acting regulatory elements in promoter regions of *TaUBPs*

**Figure 3:**
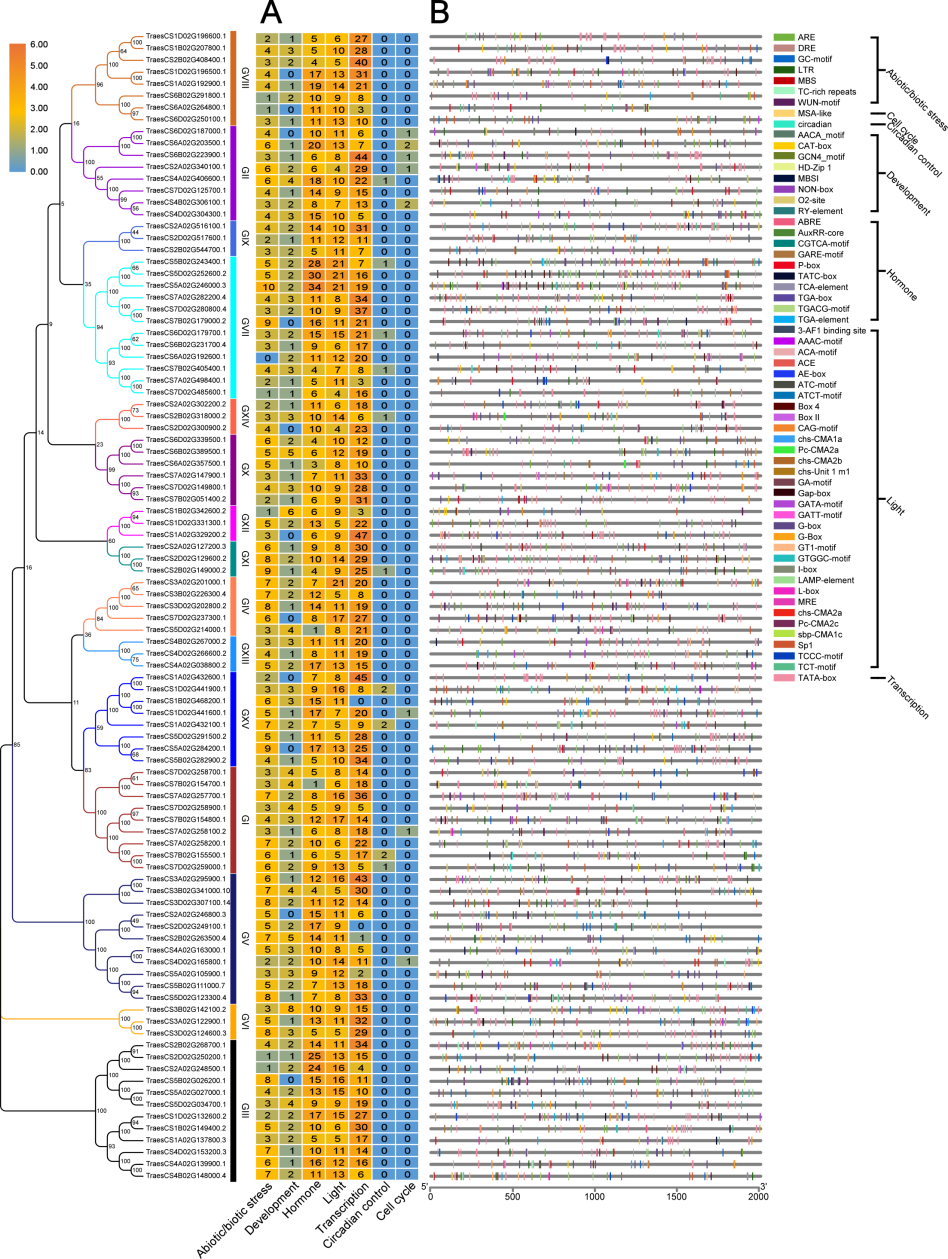
Prediction of *cis*-acting regulatory elements in *TaUBPs*. (A) The number of seven types of *cis*-acting regulatory elements detected in the promoter region of each *TaUBP*. (B) Name and position of seven types of *cis*-acting regulatory elements in *TaUBPs*.

In plants, *cis*-acting regulatory elements in the promoter regulate gene transcription by binding to target transcription factors (Butler & Kadonaga, 2002). Some *cis*-acting regulatory elements are involved in stress responses, such as hormones, dehydration, and cold responses (Liu et al., 2017; Osakabe et al., 2014; Pieterse & Van Loon, 2004; Sakuma et al., 2002). Some *cis*-acting regulatory elements are known to mediate plant immunity (Kurilla et al., 2019; Shan et al., 2016; Yu et al., 2019). The predicted *cis*-acting regulatory elements in the promoter regions of *TaUBPs* are provided in Table S4. As shown in Fig. 3, the seven types of elements related to abiotic/biotic stress, development, hormone response, light response, transcription, the circadian clock and the cell cycle are visualized. The most abundant elements were transcription-related elements (1822 in total) among the seven types of elements. In addition, there were 1044 hormone-responsive elements, 1014 light-responsive elements*,* and 436 abiotic/biotic stress-related elements. In summary, distinct *TaUBP* promoters contained different types and numbers of *cis*-acting elements.

### Tissue-specific expression of *TaUBPs*

**Figure 4:**
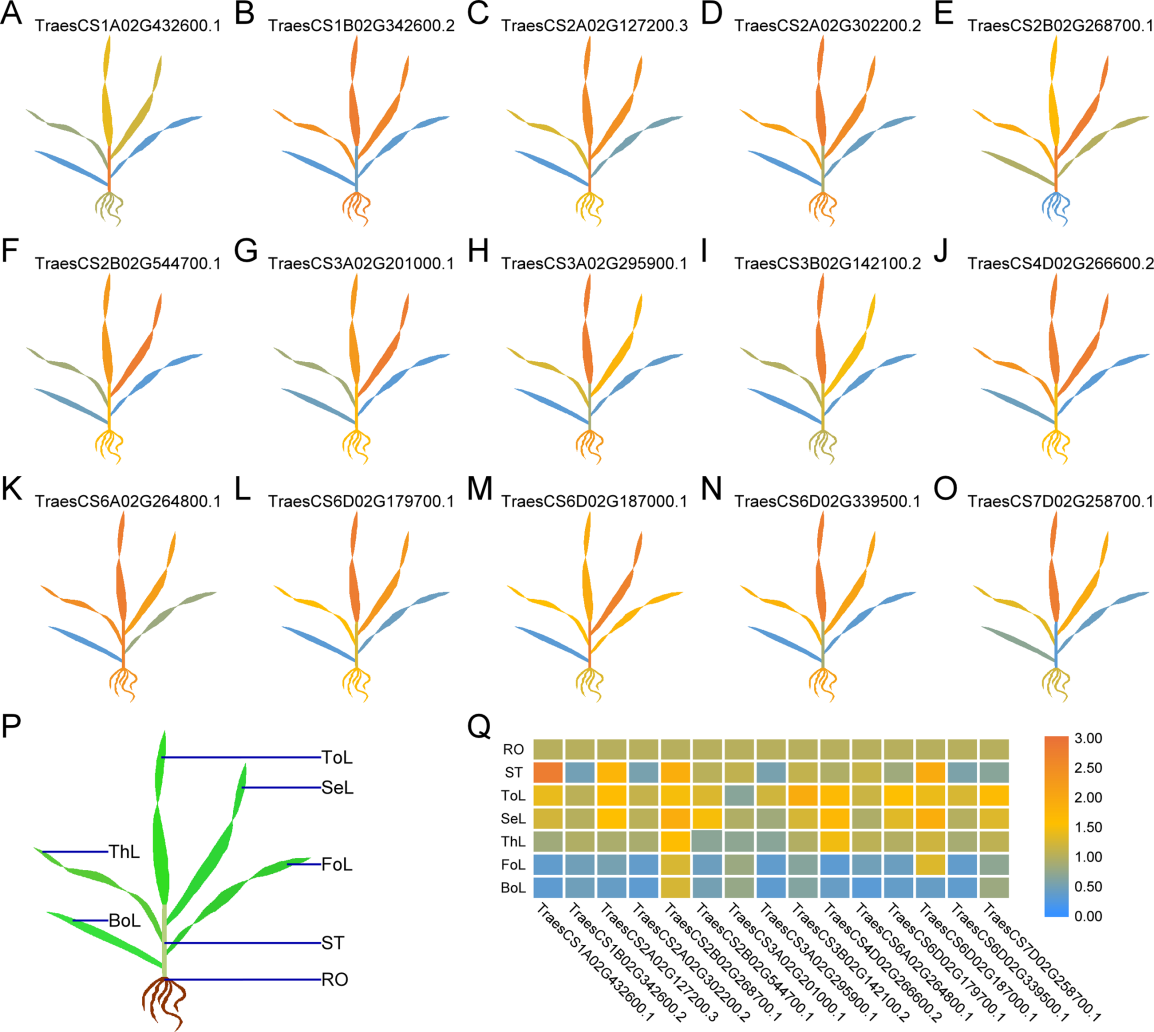
Differential expression of 15 *TaUBPs* in different tissues of wheat plants by qRT-PCR. ToL: top leaf; SeL: second leaf; ThL: third leaf; FoL: fourth leaf; BoL: bottom leaf; ST: stem; RO: root. The mean expression value was calculated from three independent biological replicates relative to the mean expression value in roots. Color scale represents log2 expression values, with the color from blue to orange indicating low to high expression abundance. The raw quantitative data of relative expression values is provided in Table S6.

To comprehensively dissect the biological functions, we randomly selected one *TaUBP* from each group as the representative for expression analysis in different tissues (roots, stems, and leaves) of wheat plants by qRT-PCR (Fig. 4). All primers used for qRT-PCR are provided in Table S5. As shown in Fig. 4, the selected 15 *TaUBPs* were expressed in all plant tissues, and the expression levels of these genes in young leaves were mostly higher than in mature ones. The *TaUBP1A.1* expression level was detected highest in stems. Followed by the *TaUBP2B.1* (*TraesCS2B02G268700.1*) which was highly expressed in stems and young leaves (top, second, and third leaf). Further *TaUBP3B.1* (*TraesCS3B02G142100.2*) and *TaUBP6D.1* (*TraesCS6D02G179700.1*) were highly expressed in the top leaf. Relative to the expression level of the *TaUBP1B.2* (*TraesCS1B02G342600.2*) in roots, the expression levels in young leaves (top, second, and third leaf) were similar. Otherwise, the expression levels of *TaUBP1B.2*, *TaUBP3A.1* (*TraesCS3A02G295900.1*) and *TaUBP6D.2* (*TraesCS6D02G339500.1*) were down-regulated in the stems relative to those in the roots.

### Expression of *TaUBPs* upon treatment with SA

**Figure 5:**
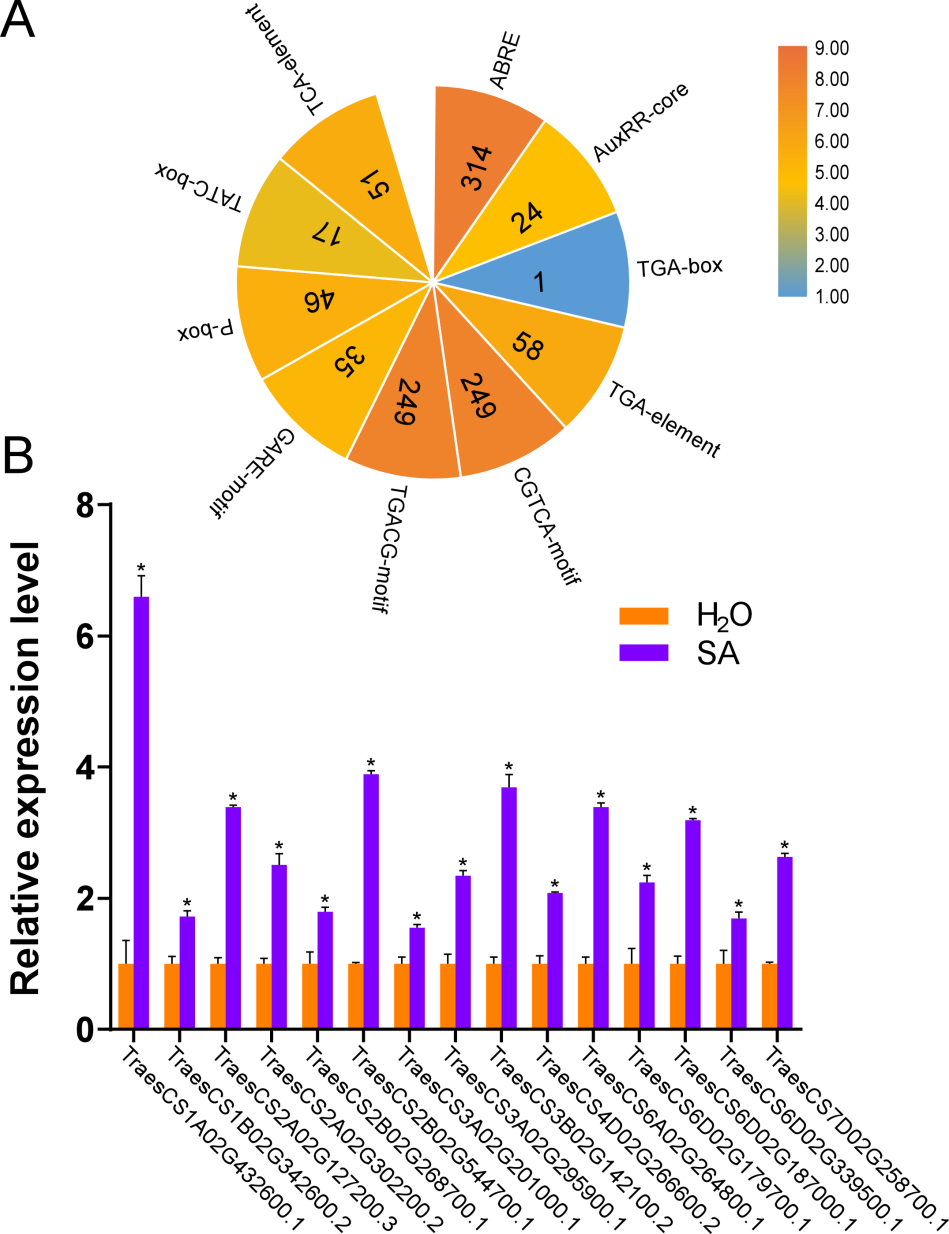
Number of hormone-responsive *cis*-acting regulatory elements in *TaUBPs* and relative expression analysis of 15 representative *TaUBPs* after three hours of SA treatment. (A) ABRE responds to ABA; AuxRR-core, TGA-box, and TGA-element respond to IAA; CGTCA-motif and TGACG-motif respond to MeJA; GARE-motif, P-box, and TATC-box respond to GA; TCA-element responds to SA. (B) Y-axes represent relative gene expression values normalized to reference gene *TaCDC*. Means and standard errors were calculated from three independent replicates. Significant differences compared with the samples water-treated were indicated by asterisks (*, P < 0.05). The raw quantitative data of relative expression values is provided in Table S7.

According to the results of the *cis*-acting regulatory elements analysis, ten types of hormone-responsive elements were identified (Fig. 5A and Table S4). As illustrated in Fig. 5A, hormone-responsive element ABREs respond to ABA; AuxRR-cores, TGA-boxes, and TGA-elements respond to auxin (IAA); CGTCA-motifs and TGACG-motifs respond to methyl jasmonate (MeJA); GARE-motifs, P-boxes, and TATC-boxes respond to Gibberellin (GA) and TCA-elements respond to SA. Among the predicted hormone-responsive elements, ABREs were the most abundant. There are 314 ABREs among hormone-responsive elements (Fig. 5A). By counting the number of certain hormone-responsive elements, the total number of MeJA-responsive elements is the most abundant. We also found TCA-elements involved in response to SA. As it has been shown that several *AtUBPs* function regulating ABA and MeJA responses, we hypothesized that the *TaUBPs* may have similar functions (An et al., 2018; Cui et al., 2013; Derkacheva et al., 2016; Zhao et al., 2016).

Hence, to explore the relationships between *UBP* genes and SA signaling, we selected 15 *TaUBPs* as representatives and performed qRT-PCR to evaluate their expression levels after SA treatment. The results showed that all selected *TaUBPs* reached peak induction and were distinctly up-regulated after three hours of SA treatment, and the most highly expressed (>6-fold that of negative controls) was *TaUBP1A.1* (Fig. 5B). In general, all selected *TaUBPs* showed significant changes in response to SA.

### Expression of *TaUBPs* under CWMV or WYMV infection

**Figure 6:**
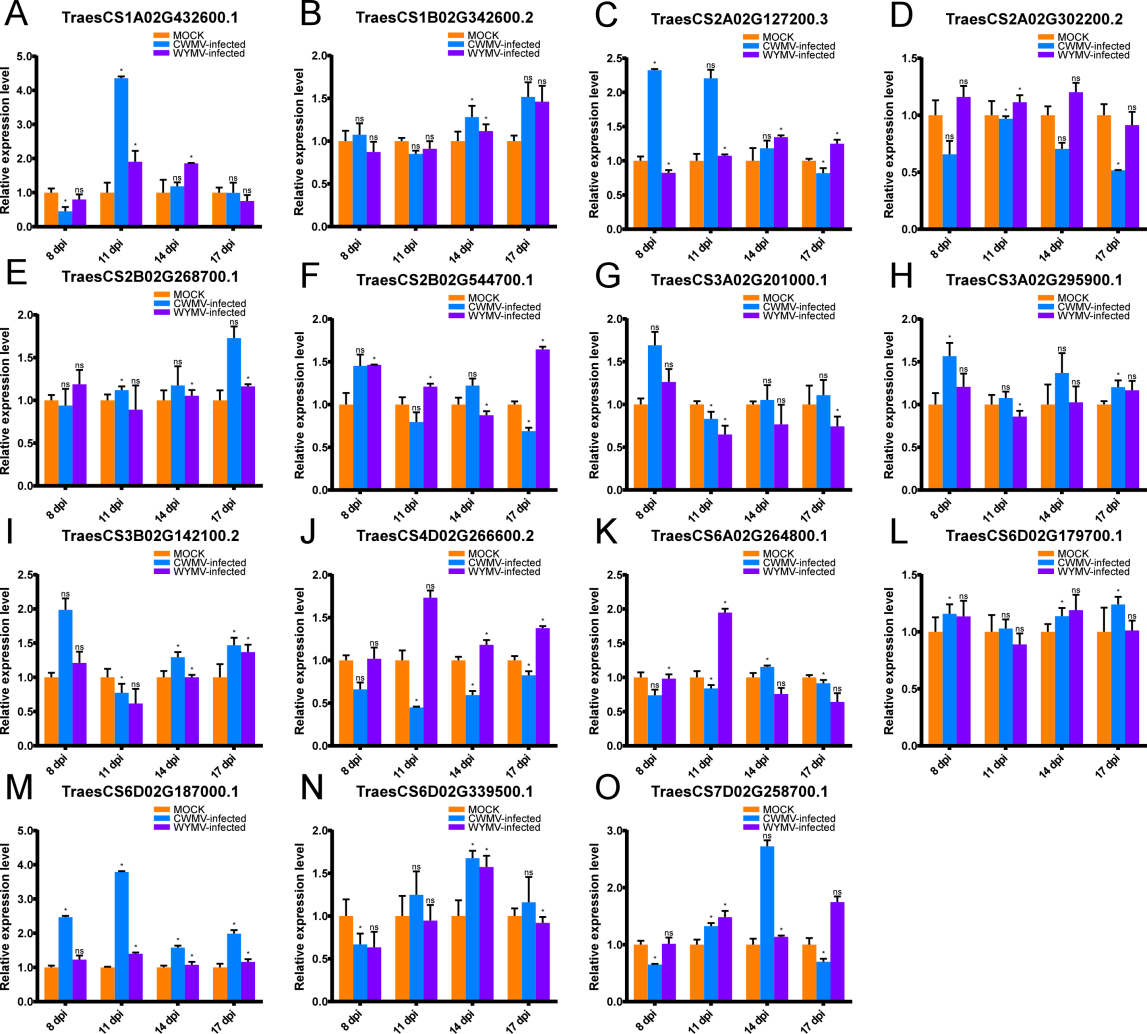
Relative expression analysis of 15 representative *TaUBPs* inoculated with CWMV or WYMV. X-axes represent time points after virus infection. Y-axes represent relative gene expression values normalized to reference gene *TaCDC*. Means and standard errors were calculated from three independent replicates. Significant differences compared with mock-inoculated controls were indicated by asterisks (*, P < 0.05). The raw quantitative data of relative expression values is provided in Table S8.

As previously mentioned, *UBPs* have multiple functions that are important for plant growth and development. However, there are no studies on *UBP* gene family members in wheat, particularly on their roles in biotic stress. CWMV and WYMV are two viruses that devastate wheat production worldwide. To investigate whether *TaUBPs* respond to CWMV or WYMV infection, wheat plants were inoculated with these two viruses. The results showed that the expression levels of 15 *TaUBPs* displayed differential induction relative to mock-inoculated controls (Fig. 6).

After CWMV infection, the expression level of *TaUBP1A.1* was distinctly up-regulated at 11 dpi, with a gradual decrease in expression at all later time points. The expression levels of the two genes *TaUBP1B.2* and *TaUBP2B.1* peaked at 17 dpi. Three genes peaked at 8 dpi, namely *TaUBP3A.1*, *TaUBP3A.2* (*TraesCS3A02G201000.1*) and *TaUBP3B.1*. Among them, the expression levels of *TaUBP3A.2* and *TaUBP3B.1* were lowest at 11 dpi, with a gradual increase at all later time points. The expression level of *TaUBP6D.2* peaked at 14 dpi, with a gradual increase during the early time points (Fig. 6).

After WYMV infection, the expression level of *TaUBP1A.1* was up-regulated at 11 and 14 dpi. *TaUBP1B.2* showed a gradual increase in expression and peaked at 17 dpi. The expression levels of *TaUBP2B.1* and *TaUBP3A.1* only exhibited slight changes over the 17-dpi time course. The expression level of *TaUBP3A.2* was down-regulated after 11 dpi. The expression level of *TaUBP3B.1* was down-regulated at 11 dpi, with a gradual increase at all later time points. The expression level of *TaUBP6D.2* was down-regulated at 8 dpi and up-regulated at 14 dpi, with a gradual increase during the early time points (Fig. 6). These results showed that most selected *TaUBPs* are substantially affected by CWMV and WYMV and may function in post-infection responses.

### Knockdown of the *TaUBP1A.1* facilitated CWMV infection in wheat

**Figure 7:**
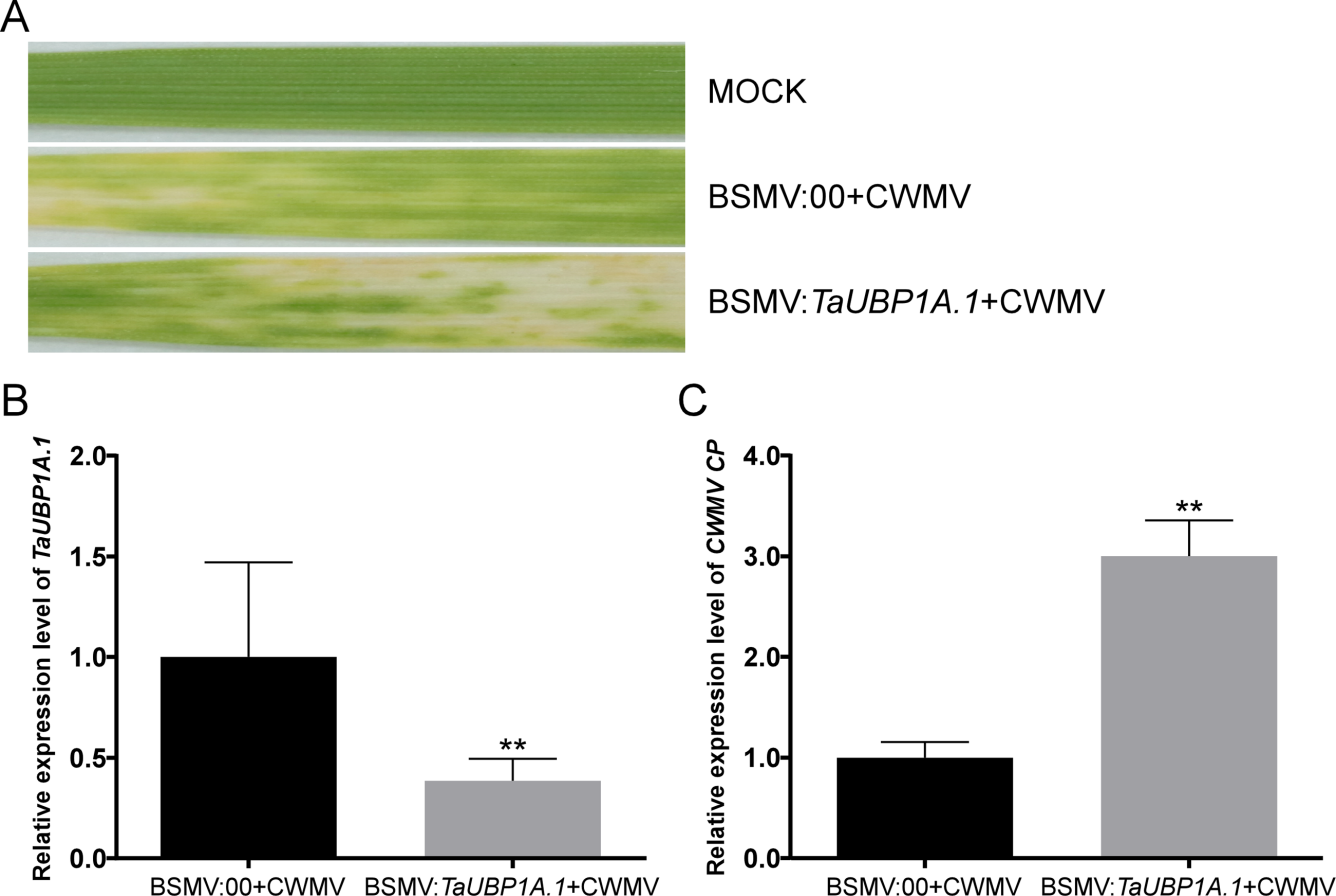
Silencing *TaUBP1A.1* through BSMV-based virus-induced gene silencing (VIGS) significantly promoted CWMV infection in *T*. *aestivum*. (A) Phenotypes in newly formed leaves of the wheat plants inoculated with FES as mock, BSMV: 00 + CWMV, BSMV: *TaUBP1A.1* + CWMV, respectively. Photographs were taken at 14 dpi. (B) Relative expression level of *TaUBP1A.1* silenced by BSMV-mediated VIGS was analyzed in *T*. *aestivum*. (C) The qRT-PCR analysis of *CWMV CP* expression in the leaves harvested from the *TaUBP1A.1*-silenced or non-silenced wheat plants. The *TaCDC* was used as the internal control. The samples from non-silenced wheat plants used as controls. Means and standard errors were calculated from three independent replicates. Significant differences were indicated by asterisks (**, P < 0.01). The raw quantitative data of relative expression values is provided in Table S9.

To investigate the relationship between *TaUBPs* expression and CWMV infection in wheat, BSMV-based virus-induced gene silencing (VIGS) was used to silence *TaUBP1A.1* in wheat. We inoculated six three-leaf-stage wheat plants with BSMV: 00 + CWMV or BSMV: *TaUBP1A.1* + CWMV. After 7 dpi, all BSMV-infected wheat plants showed mosaic symptoms in newly formed leaves, and *TaUBP1A.1*-silenced wheat plants exhibited more severe symptoms (Fig. 7A). Furthermore, we analyzed the silencing level of the *TaUBP1A.1* in the BSMV: *TaUBP1A.1* + CWMV co-inoculated wheat plants through qRT-PCR using *TaUBP1A.1* specific primers. The results demonstrated that *TaUBP1A.1* transcript level in the plants co-inoculated with BSMV: *TaUBP1A.1* + CWMV were better silenced (p <0.01) than the plants co-inoculated with BSMV: 00 + CWMV (Fig. 7B). After that, the expression level of *CWMV CP* was also detected by qRT-PCR using *CWMV CP* specific primers in these plants. The results indicated that the expression level of *CWMV CP* of BSMV: *TaUBP1A.1* + CWMV inoculated wheat was significantly higher than the inoculated wheat with BSMV: 00 + CWMV (Fig. 7C). These results suggested that silencing *TaUBP1A.1* impaired the host plant resistance to CWMV. All associated primers in this experiment are provided in Table S5.

## Discussion

The eukaryotic-specific UBP family is one of the largest families of DUBs, and it acts in plant growth and development. UBP families have been identified and characterized in several organisms, including *A. thaliana* (Yan et al., 2000), rice (Wang et al., 2018a) , Moso Bamboo (Wu et al., 2019), yeast (Wilkinson, 1997) and *M. oryzae* (Cai et al., 2020). A previous study has reported detailed characteristics and functions of *UBP* genes in *A. thaliana* (Zhou et al., 2017). However, the *UBP* gene family members have not been systematically described in wheat, and the roles of *UBP* genes in plant virus infection have not been reported. In this study, we identified and characterized 97 *UBP* genes in wheat. By analysis of two phylogenetic trees (Fig. 1, Fig. S2), *TaUBP* gene family could be classified into 15 groups (G1-G15). According to the phylogenetic tree (Fig. 1), *TaUBPs* shared high homology with *AtUBPs* and *OsUBPs*, indicating that *UBP* genes may have a relatively high homology among closely related species. This phylogenetic tree also showed that *UBPs* from the same species were distributed into different groups, revealing that *UBPs* exhibited differences in evolution among species. Additionally, *TaUBPs* existed in each group and most of the groups contained *TaUBPs*, *AtUBPs* and *OsUBPs*, revealing that *UBP* genes divergently evolved between species. As shown in Fig. S1, TaUBP proteins in each group shared a similar structure, indicating functional similarities among these proteins. Furthermore, TaUBP proteins in group G15 contain two unique domains (DUF4220 and DUF594) that do not exist in *A. thaliana* and rice. Interestingly, the UBP proteins in moso bamboo also contained special DUF4220 domains (Wu et al., 2019). The results indicated that *UBPs* in group G15 have functional similarities among wheat and moso bamboo. Based on the chromosomal location, A sub-genome contained 32 *TaUBPs*, B sub-genome contained 30 *TaUBPs* and D sub-genome contained 35 *TaUBPs*. Within all sub-genomes, the number of *TaUBPs* per chromosome varied from three to eight, manifesting *TaUBPs* non-random distribution in the chromosomes.

Gene duplication events are vital for gene expansion and help organisms adapt to various environments. By analysing the duplication relationships among *TaUBPs*, we identified two tandem duplication clusters and 54 collinear *TaUBP* gene pairs (Fig. 2). The results indicated that tandem and segmental duplication events were essential in expanding the *TaUBP* gene family and segmental duplication events seemed to be a predominant duplication pattern. To explore the evolutionary patterns of *TaUBPs*, we calculated the Ka, Ks, and Ka/Ks values for each paralogous gene (*Ta*-*Ta*) and each orthologous gene (*Ta*-*Os*). The Ks values demonstrated that duplication events occurred 1.057–68.418 Mya in wheat, and the divergence time between wheat and rice was 22.478– 73.254 Mya. In addition, the Ka/Ks ratios can be used to determine the selection mode of duplicated *UBP* genes. Here, the Ka/Ks ratios of the 54 paralogous gene pairs (*Ta*-*Ta*) were all less than one except two paralogous gene pairs were 1.001 and 1.090, suggesting that *TaUBPs* mainly underwent purification selection.

To reveal the possible biological functions of *TaUBPs*, we predicted *cis*-acting regulatory elements. According to the results, the type of *cis*-acting regulatory elements of each *TaUBP* is different. Therefore, *TaUBPs* may be involved in specific regulatory mechanisms related to various stress responses. *Cis*-acting regulatory elements largely determine tissue-specific gene expression patterns. In our study, we evaluated the expression profiles of the *TaUBPs* in different tissues (roots, stems, and leaves). According to the gene expression patterns, *TaUBPs* may be constitutively expressed in wheat plants. All selected *TaUBPs* were expressed in all tissues of the plants, and expression levels in young leaves mainly were higher than in mature leaves, suggesting that *TaUBPs* may be relative with leaf growth and development. However, faster growth and development may account for the increased expression levels observed in young leaves. A few *TaUBPs* showed tissue-specific expression in wheat. For instance, *TaUBP1B.2* showed high expression level in the roots and young leaves (top, second, and third leaf), suggesting it relates to root and leaf development. *Cis*-acting regulatory elements also largely determine stress-responsive gene expression patterns. We found an abundance of SA hormone-responsive *cis*-acting elements in the promoter regions of *TaUBPs*, indicating the essential roles of *TaUBPs* in SA hormone-stress responses. Accordingly, we explored the relationships between *TaUBPs* and SA signaling by validating the expression levels of *TaUBPs* after SA treatment. After three hours of SA treatment, 15 analyzed *TaUBPs* were distinctly up-regulated and peaked at this time point. Overall, *TaUBPs* showed significant changes, implying that several *TaUBPs* have potential functions in response to SA.

To investigate whether *TaUBPs* are responsive to viruses, wheat plants were inoculated with CWMV or WYMV. Our results showed that relative to mock-inoculated controls, the expression levels of most of the *TaUBPs* that were analyzed were substantially altered after infection. For instance, we found that the expression level of *TaUBP1A.1* reached the highest level at 11 dpi, with a gradual decrease at all later time points after CWMV infection. As mentioned above, studies have reported that *UBPs* are involved in pathogen defense and immune response. Therefore, we hypothesized that *TaUBPs* might respond to viral infection. Subsequently, we used BSMV-mediated VIGS to transiently silence the *TaUBP1A.1* to investigate its biological function after CWMV infection. The experiment data showed the expression level of *CWMV CP* in *TaUBP1A.1*-silenced wheat plants was significantly increased compared to the non-silenced wheat plants, suggesting that *TaUBP1A.1* responses to CWMV infection in wheat. Thus, *UBP* gene family members in wheat might possess specific functions in defensing viruses.

## Conclusion

In this study, 97 *TaUBPs* were identified and characterized. We constructed two phylogenetic trees and systematically analyzed evolutionary and divergence patterns alongside the stress responses of selected *TaUBPs*. These *TaUBPs* could be divided into 15 groups, and the members of *TaUBP* gene family from the same group were found to have a similar protein structure. The divergence time of *UBP* genes indicated a complex evolutionary history for this family in wheat. The expression profiles of *UBP* genes indicated that these genes play a crucial role in plant growth and development, as well as in stress responses. In addition, silencing *TaUBP1A.1* enhanced the infection of wheat by CWMV. This is a systematic and comprehensive study of *TaUBPs* that can aid in cloning and functional analyses in wheat and lays the foundation for further exploration of the relationship between *UBPs* and immunity in this important crop.

##  Supplemental Information

10.7717/peerj.11594/supp-1 Supplemental Information 1 Phylogenetic relationships and conserved domains in TaUBP proteins

10.7717/peerj.11594/supp-2 Supplemental Information 2 The phylogenetic tree based on 97 TaUBP protein sequences

10.7717/peerj.11594/supp-3 Supplemental Information 3 The detailed information about 97 predicted *Ta UBPs*

10.7717/peerj.11594/supp-4 Supplemental Information 4 Ka, Ks and Ka/Ks values calculated for paralogous and orthologous gene pairs

10.7717/peerj.11594/supp-5 Supplemental Information 5 The detailed information of the instruments and the reagents

10.7717/peerj.11594/supp-6 Supplemental Information 6 The *cis*-acting regulatory elements in the promoter regions of *TaUBP s*

10.7717/peerj.11594/supp-7 Supplemental Information 7 All primers used for this study

10.7717/peerj.11594/supp-8 Supplemental Information 8 The raw quantitative data for Fig. 4

10.7717/peerj.11594/supp-9 Supplemental Information 9 The raw quantitative data for Fig. 5

10.7717/peerj.11594/supp-10 Supplemental Information 10 The raw quantitative data for Fig. 6

10.7717/peerj.11594/supp-11 Supplemental Information 11 The raw quantitative data for Fig. 7

## Additional Information and Declarations

### Data Availability

The following information was supplied regarding data availability: The raw data are available in the Supplementary Files.
