# Supplementary figures and images for: Genome-wide identification and characterization of UBP gene family in wheat (Triticum aestivum L.)
Source: PeerJ. 2021 Jun 15;9:e11594. doi: 10.7717/peerj.11594 (PMC8212830; doi:10.7717/peerj.11594)

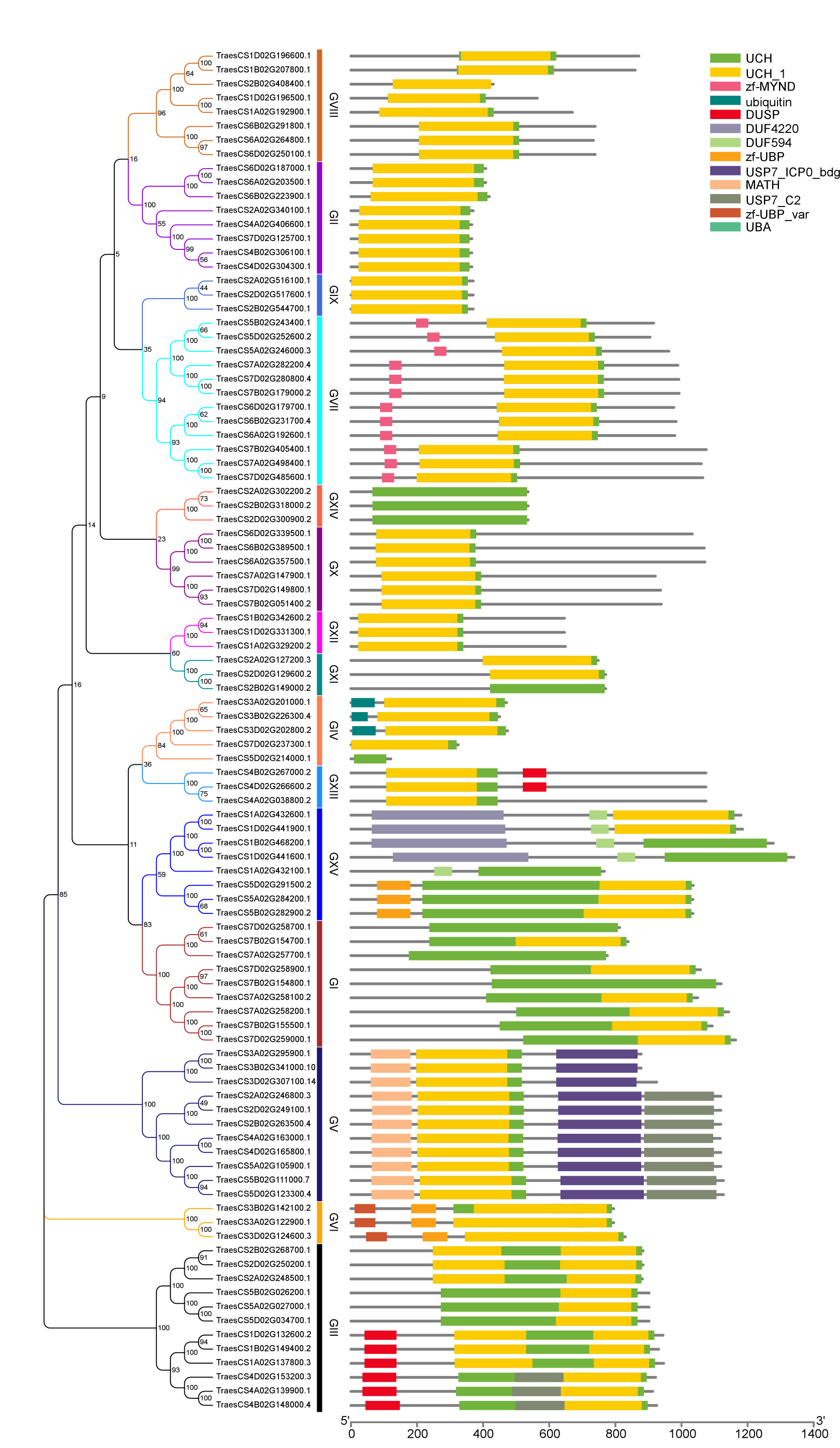

Supplement: Supplemental Information 1 [file peerj-09-11594-s001.png]

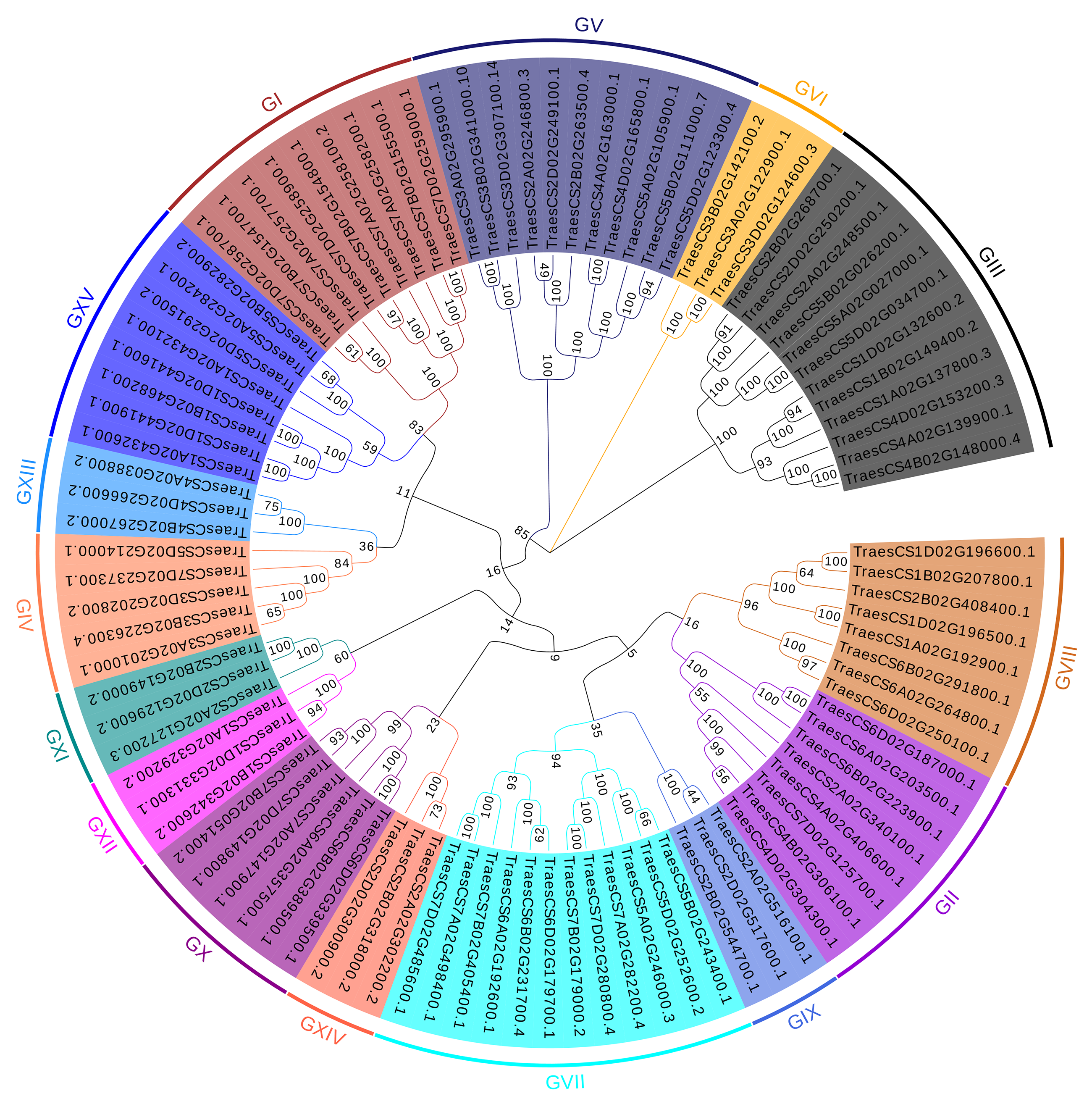

Supplement: Supplemental Information 2 [file peerj-09-11594-s002.png]
